# Pathometagenomics reveals susceptibility to intestinal infection by Morganella to be mediated by the blood group-related B4galnt2 gene in wild mice

**DOI:** 10.1080/19490976.2022.2164448

**Published:** 2023-01-22

**Authors:** Marie Vallier, Abdulhadi Suwandi, Katrin Ehrhardt, Meriem Belheouane, David Berry, Aleksa Čepić, Alibek Galeev, Jill M. Johnsen, Guntram A. Grassl, John F. Baines

**Affiliations:** a Section of Evolutionary Medicine, Institute for Experimental Medicine, Kiel University, Kiel, Germany; b Guest Group Evolutionary Medicine, Max Planck Institute for Evolutionary Biology, Plön, Germany; c Institute of Medical Microbiology and Hospital Epidemiology, Hannover Medical School, Hannover, Germany; d German Center for Infection Research (DZIF), Hannover, Germany; e Centre for Microbiology and Environmental Systems Science, Department of Microbiology and Ecosystem Science, Division of Microbial Ecology, University of Vienna, Vienna, Austria; f Joint Microbiome Facility of the Medical University of Vienna and the University of Vienna, Vienna, Austria; g Bloodworks Research Institute, Seattle, WA, USA; h Department of Medicine, University of Washington, Seattle, WA, USA

**Keywords:** *Morganella*, enteric infection, *B4galnt2*, balancing selection, blood group, gut microbiome, wild mice

## Abstract

Infectious disease is widely considered to be a major driver of evolution. A preponderance of signatures of balancing selection at blood group-related genes is thought to be driven by inherent trade-offs in susceptibility to disease. B4galnt2 is subject to long-term balancing selection in house mice, where two divergent allele classes direct alternative tissue-specific expression of a glycosyltransferase in the intestine versus blood vessels. The blood vessel allele class leads to prolonged bleeding times similar to von Willebrand disease in humans, yet has been maintained for millions of years. Based on in vivo functional studies in inbred lab strains, it is hypothesized that the cost of prolonged bleeding times may be offset by an evolutionary trade-off involving susceptibility to a yet unknown pathogen(s). To identify candidate pathogens for which resistance could be mediated by B4galnt2 genotype, we here employed a novel “pathometagenomic” approach in a wild mouse population, which combines bacterial 16S rRNA gene-based community profiling with histopathology of gut tissue. Through subsequent isolation, genome sequencing and controlled experiments in lab mice, we show that the presence of the blood vessel allele is associated with resistance to a newly identified subspecies of Morganella morganii, a clinically important opportunistic pathogen. Given the increasing importance of zoonotic events, the approach outlined here may find useful application in the detection of emerging diseases in wild animal populations.

## Introduction

Infectious disease is widely considered to be a major driver of evolution^1^. Genes involved in immune defense display elevated rates of change between species and studies of the genetics of speciation reveal evidence of a large immune effect.^2^ Similarly, the impact of pathogen-driven selective pressure can also be reflected by patterns of genetic variation within species, such as selective sweeps and/or balancing selection associated with resistance mutations.^3–5^

In the case of balancing selection, the maintenance of genetic variants mediating resistance to pathogens may be associated with inherent tradeoffs.^6^ In some cases, such balanced polymorphisms can be maintained beyond species boundaries, known as trans-species polymorphism, and are taken as evidence of long-term balancing selection.^7^ In particular, systematic screens for balancing selection in the human genome reveal genes mainly involved in host–pathogen interaction.^3,8,9^ Notable examples in humans and other mammals include the MHC locus^10^ and blood group-related gene,^11–13^ where the importance of the latter is most recently exemplified by the ongoing COVID-19 pandemic.^14,15^ That the maintenance of genetic variants at these loci involves tradeoffs is supported by association studies implicating them in a vast number of genetic diseases,^11,16^ although the individual pathogens involved are often less clear.

We previously showed that the blood-group related glycosyltransferase gene *B4galnt2* (Beta-1,4 N-acetyl galactosaminyl transferase 2) displays strong signatures of long-term balancing selection in wild house mice,^17,18^ as well as signs of more recent selection in the form of a partial selective sweep within local *Mus musculus domesticus* populations from south-west France.^19^ Based on *in vivo* functional studies in inbred lab strains, these dynamics likely result from a trade-off between the cost of prolonged bleeding times^20^ and susceptibility to a yet unknown pathogen(s).^21,22^ Accordingly, two highly divergent alleles of *B4galnt2* display *cis*-regulatory variation in their tissue-specific expression patterns. While the wild-type allele class found in the C57BL/6J and other mouse strains drives gastrointestinal epithelial expression, an alternative allele class originally discovered in the RIIIS/J strain exhibits a loss of intestinal expression and instead drives vascular endothelial expression, leading to a phenotype similar to the human bleeding disorder von Willebrand disease.^20,23^ The prolonged bleeding times in RIIIS/J are attributable to very low levels of the endothelial-expressed coagulation protein von Willebrand factor (VWF) caused by aberrant glycosylation by *B4galnt2* and accelerated clearance.^20^ In C57BL/6J mice, we previously demonstrated that gastrointestinal expression of *B4galnt2* influences resident microbes, most likely due to the presentation of the *B4galnt2*-specific GalNAc on mucosal surfaces,^24^ in addition to influencing susceptibility to experimental *Salmonella* infection.^22^

In this study, we used a combination of pathology and metagenomics (i.e. “pathometagenomics”) to reveal candidate pathogens potentially contributing to selection at *B4galnt2* in a wild house mouse population. By sampling over 200 animals from south west France, we show that *B4galnt2* genotype correlates with gastrointestinal inflammation, and candidate pathogens are more prominent in inflamed C57BL/6J-class homozygotes. Finally, we experimentally demonstrate that *B4galnt2* plays a significant role in the severity of infection with a candidate pathogen belonging to a new subspecies of *Morganella morganii*, a clinically important pathogen in humans. Given the recent importance of zoonotic events in triggering global pandemics, the approach outlined here may find useful application in the monitoring of emerging diseases in wild animal populations.

## Results

### Wild mouse sampling, population structure, and B4galnt2 genotyping

In order to investigate the possible link between *B4galnt2* genotype and susceptibility to gastrointestinal pathogens in the wild, we sampled wild house mice at a location in southwestern France, which was chosen based on previous evidence of selection at this locus.^19,25^ In total, 217 animals were collected from 34 farms (*Suppl*. Tables 1 & 2), which were first described in another study by our group.^26^

**Figure 1.**
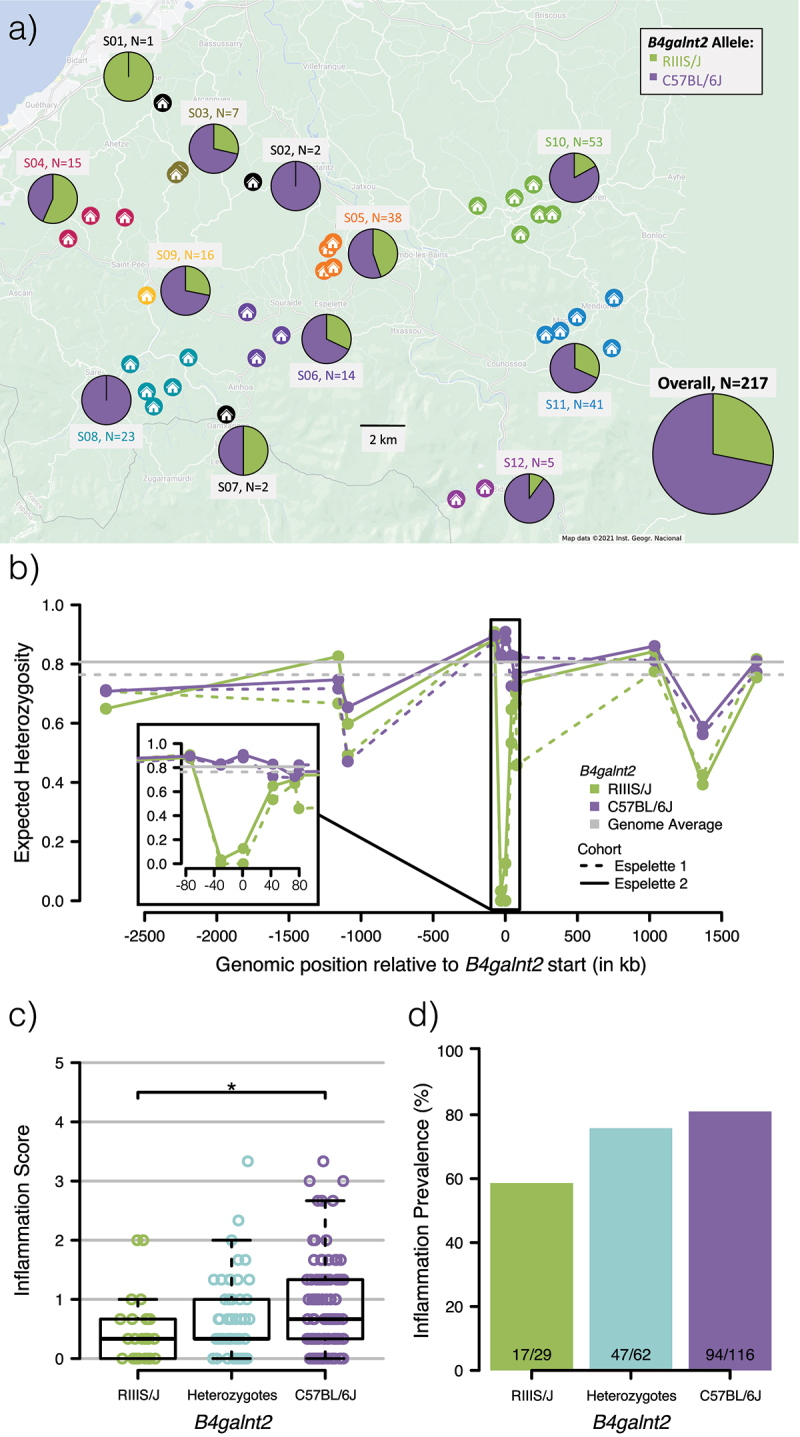
**Geographic distribution of *B4galnt2* alleles, linked variation and inflammation. a)** *B4galnt2* allele frequency at 12 super families sampled in southwest France and overall. For each super family, the identifier (S##) and sample size are shown. **b)** Expected heterozygosity at 12 *B4galnt2*-linked microsatellite loci, phased according to *B4galnt2* haplotypes. Previously published data (Espelette 1)^19^ as well as data from this study (Espelette 2) are presented. **c-d)** Inflammation score (c) and prevalence (d) derived from cecum histology according to *B4galnt2* genotype. Pairwise Wilcoxon (c) and pairwise χ^2^ (d) tests were used with “FDR” correction for multiple testing (* p < .05).

**Figure 2.**
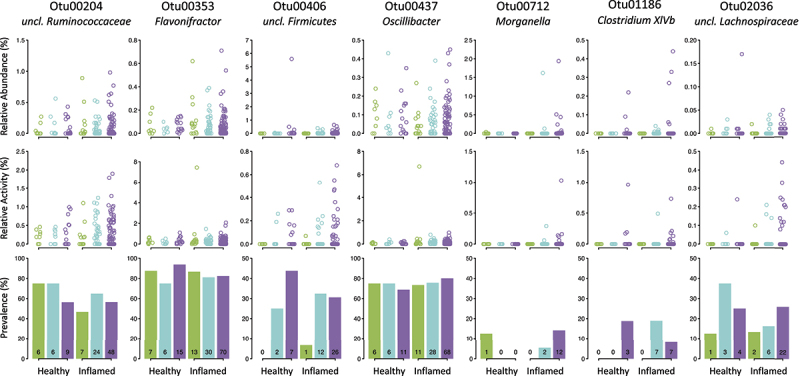
**Distribution of 7 candidate pathogens according to *B4galnt2* genotype and cecum inflammation**. Relative abundance, relative activity, and prevalence of candidate pathogens according to cecum inflammation (healthy/inflamed) and *B4galnt2* genotype (green = RIIIS/J homozygotes; turquoise = heterozygotes; purple = C57BL/6J homozygotes). Counts are written in the prevalence bar graphs.

**Figure 3.**
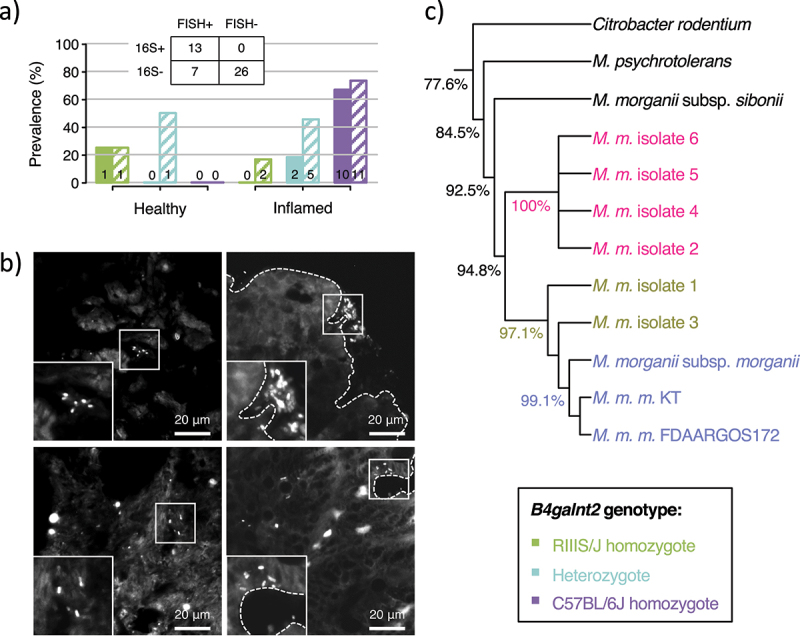
**Investigation of the candidate pathogen *Morganella* in wild mice**. a) Prevalence of *Morganella* detected in the cecum of wild mice by 16S rRNA analysis (filled bars) and FISH analysis (striped bars) for a subset of 46 samples, with the corresponding contingency table. Data are split according to inflammation prevalence (healthy/inflamed) and *B4galnt2* genotype (green = RIIIS/J homozygotes; turquoise = heterozygotes; purple = C57BL/6J homozygotes). **b)** Fluorescence in situ hybridization targeting *Morganella* in the cecum of wild mice show signal in the lumen (left) and the tissue (right). **b)** Neighbor-joining phylogenetic tree of the six isolates of *Morganella morganii* obtained from wild samples, as well as three *Morganella* type strains and two publicly available genomes of *Morganella morganii morganii* strains. *Citrobacter rodentium* was used as outgroup. Percentages indicate the mean pairwise identity between branches.

**Figure 4.**
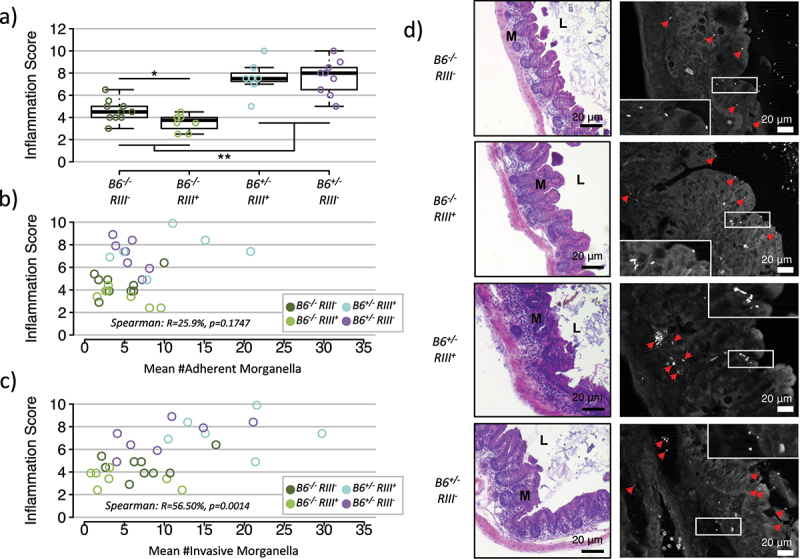
**Experimental infection with *Morganella* in lab mice. a)** Inflammation score in the cecum of C57BL/6J mice experimentally infected with *Morganella* according to *B4galnt2* genotype/expression category (pairwise Wilcoxon test with “FDR” correction for multiple testing; ** p < .01, * p < .05). **b-c)** Inflammation score in the cecum of C57BL/6J mice experimentally infected with *Morganella* with respect to the mean number of adherent (b) and invasive (c) *Morganella* detected in at least 10 fields of view by FISH. Positive signal was not counted beyond 30. **d)** Representative pictures of H&E staining and FISH.

To account for demographic factors that could possibly confound associations between *B4galnt2* genotype and candidate pathogens, we (i) collected mitochondrial D-loop sequences, (ii) typed 18 neutral microsatellite loci,^25^ and (iii) clustered farms into families and super families based on geographic distance (Figure 1a, *Suppl. Table 3*), following previously suggested guidelines to take local familial/inbreeding patterns into account.^27^ The mitochondrial D-loop sequences confirm that all animals belong to *M. m. domesticus*, and that our sample displays similar mitochondrial haplogroup structure to that observed in a previous survey of Western Europe (*Suppl*. Figure 1).^25^ The 18 neutral microsatellite data (*Suppl. Table 4*) was used as input for STRUCTURE analysis to identify clusters of ancestry, allowing for admixture. We find our regional sampling to be composed of 16 distinct clusters, thereafter referred as “populations”, whereby five have admixed ancestry (*Suppl*. Figure 2 *& Suppl. Table 5*). In addition, we calculated the pairwise relatedness between all animals using KINGROUP (*Suppl. Table 6*). This reveals that mice coming from the same super family tend to be more related than those coming from distinct locations (>3.5 km apart; see Methods), consistent with known local inbreeding effects in mice (*Suppl*. Figure 3). In particular, we observe a strong correspondence between relatedness, super family, and population, while mitochondrial haplogroups are more dispersed across locations and populations (*Suppl*. Figures 3 & 4). This information was subsequently used in a comprehensive statistical model described below, which is designed to detect candidate pathogens according to host genotype while accounting for these demographic parameters.

To obtain *B4galnt2* genotype information, we sequenced a diagnostic PCR fragment,^18^ which identified 125 mice as C57BL/6J  homozygotes, 62 as heterozygotes, and 30 as RIIIS/J homozygotes (*Suppl*. Table 2). This corresponds to an overall RIIIS/J allele frequency of 28%, which is comparable to the 36% previously observed at this sampling location (Espelette).^19^ Importantly, both allele classes are present in a majority (9/12) of super families (Figure 1a, *Suppl*. Figure 3), which allows for the downstream statistical analysis according to *B4galnt2* genotype to be on average nested within the multiple local environments of individual farms/super families.

Further, as we previously observed evidence of a partial selective sweep on the background of the RIIIS/J allele in Southern France based on multiple sampling trips and field sites,^17,19^ we typed an additional 12 microsatellites across the *B4galnt2* gene region in the current sampling to confirm and characterize allele-specific dynamics. After determining the haplotypic phase of diploid individuals according to *B4galnt2* allele class (*Suppl. Table 7*), we again observe a drastic reduction in heterozygosity on the background of the RIIIS/J allele, which approaches zero at the two loci closest to the upstream region of *B4galnt2* known to contain a cis-regulatory mutation responsible for alternative tissue-specific expression patterns (Figure 1b).^20^ These results thus confirm our previous evidence of a partial allele-specific sweep, and indicate recent selection favoring an increase in the RIIIS/J allele class in this region.

### Inflammation differs according to B4galnt2 genotype

In order to test the hypothesis that variation in *B4galnt2* expression mediates differences in susceptibility to pathogens in the wild, we examined intestinal (cecum) tissue for the presence of inflammation as an indicator of possible ongoing infection using histological analysis (see Methods). An inflammation score was calculated for each mouse based on the sum of levels of epithelial desquamation, necrosis, and infiltration of polymorphonuclear leukocytes in the mucosa and the submucosa (n = 207 after excluding inadequately preserved samples; *Suppl. Table 8*). Interestingly, a significant association is observed according to *B4galnt2* genotype, whereby individuals homozygous for the RIIIS/J allele class display the lowest inflammation scores, which are significantly lower than the scores of C57BL/6J homozygotes (pairwise Wilcoxon test; p = .015) (Figure 1c). Heterozygous individuals appear to have an intermediate phenotype, but do not significantly differ from either homozygote (p = .13 and p = .14). A similar trend is observable with the prevalence of inflammation (Figure 1d), with 60% of RIIIS/J homozygotes showing inflammation compared to 80% of C57BL/6J homozygotes (pairwise χ^2^ test; p = .06). This pattern is consistent with the hypothesis that the RIIIS/J allele could provide protection against intestinal pathogens. To independently verify our inflammation scoring, we also measured the expression of three inflammatory cytokines including Interleukin 1 beta (*Il1b*), Interferon gamma (*Ifng*), and Monocyte chemoattractant protein 1 (*Mcp1*) via quantitative PCR (qPCR). The expression of *Ifng* and *Mcp1* display a significant positive correlation to the inflammation score based on histological scoring (*Il1b* rho = 0.098, p = .160; *Ifng* rho = 0.298, p < .001; *Mcp1* rho = 0.147, p = .036), although no individual gene significantly differed according to *B4galnt2* genotype (*Suppl. Figure 5*).

### Culture-independent bacterial community analysis

To evaluate the potential relationship between *B4galnt2* genotype, cecum pathology, and intestinal microbial populations, we performed 16S rRNA gene sequencing of the cecal tissue-associated microbiota. To capture patterns that may be differentially revealed by bacterial cell number or activity, sequencing was performed at the DNA and the RNA (cDNA) levels, respectively. DNA-based data is accordingly referred to as “abundance”, while RNA-based data was normalized to DNA and referred to as “activity”. A composite prevalence was computed by counting OTUs as present if detected in either the abundance or activity dataset. After applying all quality filtering criteria (see Methods) in addition to including only samples for which histological assessment was possible, a total of 169 samples were used for further analysis.

Dysbiosis is widely described in humans suffering from intestinal inflammation as well as in a variety of animal models. This phenomenon is generally associated with an overall alteration of microbial community composition and reduced diversity.^28^ Therefore, we first evaluated whether overall community-level patterns (alpha- and beta diversity) are present according to *B4galnt2* genotype and/or inflammation status in our wild mouse cohort, taking experimental variables (extraction batch, sequencing library) and mouse demographic parameters/metadata (super family, population, mitochondrial haplogroup, weight) into account. No relationship is observed between alpha diversity (Chao and inverse Simpson index) and inflammation or genotype (*Suppl. Table 9*). In contrast, a significant correlation between overall microbial community composition (beta diversity as determined by the Bray-Curtis and Jaccard indices) is observed with respect to inflammation score (Table 1). Although the association is weak (effect size lower than 1%), it is noteworthy that the effect is significant only when based on the activity of community members, but not their relative abundance. This suggests that a small portion of the bacterial community is disproportionally active in inflamed mice, which could be consistent with pathogenic behavior.

**Table 1.** Beta diversity with respect to experimental, demographic, and mouse variables. Effect sizes and FDR corrected p-values of PERMANOVA tests between Bray-Curtis (Abundance and Activity) or Jaccard (Prevalence) distances and mouse characteristics, added sequentially in the final adonis models. Confounding variables were added sequentially in all models, in the order presented in the table from left to right, variables of interest were added independently in distinct models.

		*Confounding variables*						*Variables of Interest*		
		Extraction	Library	Super Family	Population	Haplogroup	Weight	*B4galnt2*	Inflammation Score	Inflammation Prevalence
Abundance (DNA)	**effect size**	**12.03%**	**1.67%**	**7.48%**	**9.23%**	2.52%	**0.83%**	0.93%	0.77%	0.44%
	**p-value**	**0.0001**	**0.0022**	**0.0002**	**0.0001**	0.0532	**0.0198**	0.8272	0.1715	0.8272
Activity (RNA)	**effect size**	**12.68%**	**1.38%**	**7.29%**	**8.57%**	**2.54%**	0.67%	1.00%	**0.87%**	0.60%
	**p-value**	**0.0001**	**0.0305**	**0.0007**	**0.0007**	**0.0409**	0.1034	0.6867	**0.0465**	0.4153
Prevalence	**effect size**	**12.57%**	**1.52%**	**7.72%**	**8.85%**	**2.58%**	**0.73%**	0.98%	0.84%	0.47%
	**p-value**	**0.0001**	**0.0108**	**0.0003**	**0.0003**	**0.0282**	**0.0372**	0.7960	0.0555	0.7960

### Candidate pathogen detection

In order to identify individual bacterial taxa that could be linked to the maintenance of variation at *B4galnt2* in wild populations, individual OTUs were evaluated with respect to *B4galnt2* genotype, cecum inflammation, and their interaction, using a comprehensive linear model framework that accounts for the potential confounding demographic factors described above (see Methods). The models were evaluated on the levels of taxon abundance, activity, and prevalence, and candidates were selected only when the overall model, *B4galnt2* genotype, and inflammation reached significance. This analysis reveals seven candidate OTUs (Table 2, Figure 2), four of which show an association to *B4galnt2* genotype and inflammation at the presence/absence level (Otu01186, 02036, 00204, 00406) and two that show an association to *B4galnt2* genotype, inflammation, and their interaction at the activity level (Otu00353, 00437). A final interesting candidate (Otu00712) is identified by two models including (i) *B4galnt2* genotype, inflammation, and their interaction at the abundance level and (ii) *B4galnt2* genotype and inflammation at the presence/absence level, in addition to being significant for inflammation alone at the activity level. For further characterization and validation, we chose Otu00712 for several reasons. First, it belongs to the genus *Morganella*, a known opportunistic pathogen in the *Enterobacteriaceae* family. Second, it is nearly entirely absent from non-inflamed animals, being present in only a single non-inflamed RIIIS/J homozygote, but 14 inflamed animals carrying one or more copies of the C57BL/6J allele, and is thus consistent with a higher pathogenicity in mice expressing *B4galnt2* in the gastrointestinal tract (Figure 2). In contrast, most other candidates are also present in appreciable numbers in non-inflamed mice and/or belong to taxa that are typical core commensals among wild mouse communities,^25^ and thus do not appear to display fully consistent pathogenic properties.

**Table 2.** Candidate OTUs associated with *B4galnt2* and inflammation. For each OTU, the p-value and effect size (%) of the association with every parameter in the model is given, as well as the overall p-value and effect size (%) of the model. Stars denote significant associations to both *B4galnt2* genotype and inflammation.

OTU#		Library		Super Family		Population		Haplogroup		Weight		*B4galnt2*		Inflammation		Interaction		Overall	
***Relative Abundance***																			
Otu01186		0,709	0,42%	0,964	2,48%	0,130	11,66%	0,281	3,09%	0,097	1,68%	0,584	0,65%	0,497	0,28%	0,900	0,13%	0,574	20,38%
Otu02036		0,135	2,43%	0,179	9,20%	0,917	3,94%	0,671	1,41%	0,963	0,00%	0,058	3,48%	0,284	0,69%	0,951	0,06%	0,499	21,21%
Otu00353		0,054	3,40%	0,979	2,04%	0,413	7,73%	**0,023**	**6,67%**	0,591	0,17%	0,261	1,54%	0,640	0,13%	0,064	3,20%	0,215	24,87%
Otu00437		0,233	1,47%	**0,027**	**11,37%**	0,915	3,31%	**0,000**	**15,85%**	0,398	0,36%	0,981	0,02%	0,314	0,51%	0,283	1,27%	**0,005**	**34,16%**
Otu00204		0,371	1,07%	0,648	4,66%	0,056	12,27%	**0,006**	**8,04%**	0,094	1,52%	0,748	0,31%	0,219	0,82%	0,552	0,64%	**0,046**	**29,33%**
Otu00406		**0,000**	**1,19%**	**0,000**	**11,31%**	**0,000**	**83,84%**	**0,024**	**0,29%**	0,728	0,00%	0,248	0,07%	0,414	0,02%	0,676	0,02%	**0,000**	**96,74%**
**Otu00712**	*	0,766	0,28%	0,403	6,00%	0,304	7,90%	0,809	0,83%	0,092	1,49%	**0,008**	**5,12%**	**0,006**	**3,97%**	**0,003**	**6,14%**	**0,016**	**31,73%**
***Relative Activity***																			
Otu01186		0,403	0,91%	0,261	6,80%	**0,033**	**12,36%**	0,762	0,92%	**0,000**	**7,33%**	0,088	2,45%	**0,010**	**3,41%**	0,597	0,51%	**0,004**	**34,68%**
Otu02036		**0,038**	**4,16%**	0,497	6,46%	0,984	2,75%	0,941	0,48%	0,862	0,02%	0,056	3,65%	0,355	0,53%	0,893	0,14%	0,757	18,20%
**Otu00353**	*	0,413	0,88%	0,955	2,17%	0,499	6,16%	0,547	1,53%	0,266	0,62%	**0,015**	**4,32%**	**0,011**	**3,32%**	**0,000**	**15,53%**	**0,004**	**34,52%**
**Otu00437**	*	0,544	0,56%	0,961	1,94%	0,844	3,64%	0,576	1,34%	0,175	0,85%	**0,005**	**5,10%**	**0,006**	**3,55%**	**0,000**	**22,26%**	**0,000**	**39,24%**
Otu00204		0,590	0,53%	0,256	6,95%	**0,000**	**20,49%**	0,945	0,38%	0,718	0,07%	0,105	2,30%	0,322	0,50%	0,093	2,43%	**0,006**	**33,65%**
Otu00406		**0,049**	**3,52%**	0,513	5,87%	0,366	8,17%	0,964	0,34%	0,415	0,38%	**0,017**	**4,78%**	0,148	1,21%	0,891	0,13%	0,244	24,41%
Otu00712		**0,044**	**3,44%**	0,120	9,19%	0,104	10,92%	0,960	0,34%	0,066	1,85%	0,877	0,14%	**0,041**	**2,28%**	0,439	0,89%	0,052	29,05%
***Presence/Absence***																			
**Otu01186**	*	0,610	0,44%	0,553	4,37%	**0,001**	**16,76%**	0,155	3,03%	**0,000**	**6,43%**	**0,000**	**7,61%**	**0,023**	**2,38%**	0,996	0,01%	**0,000**	**41,02%**
**Otu02036**	*	**0,016**	**4,39%**	0,081	9,64%	0,148	9,72%	0,596	1,44%	0,320	0,52%	**0,043**	**3,33%**	**0,031**	**2,46%**	0,848	0,17%	**0,016**	**31,67%**
Otu00353		0,391	1,04%	0,136	9,06%	0,623	5,93%	**0,012**	**7,30%**	0,126	1,30%	0,763	0,30%	**0,046**	**2,22%**	0,543	0,67%	0,083	27,82%
Otu00437		0,066	3,28%	0,285	7,88%	0,724	5,66%	0,446	2,21%	0,250	0,79%	0,835	0,21%	0,246	0,80%	0,366	1,20%	0,428	22,02%
**Otu00204**	*	0,517	0,61%	**0,017**	**11,17%**	**0,006**	**14,41%**	0,463	1,67%	0,089	1,35%	**0,017**	**3,86%**	**0,001**	**5,25%**	0,330	1,03%	**0,000**	**39,34%**
**Otu00406**	*	0,063	2,82%	0,346	6,19%	**0,007**	**15,33%**	0,181	3,18%	0,833	0,02%	**0,015**	**4,34%**	**0,048**	**2,00%**	0,928	0,08%	**0,005**	**33,96%**
**Otu00712**	*	0,897	0,10%	**0,023**	**10,95%**	**0,002**	**16,67%**	0,620	1,24%	0,624	0,11%	**0,005**	**5,24%**	**0,007**	**3,49%**	0,704	0,33%	**0,000**	**38,13%**

### New subspecies of Morganella morganii with pathogenic potential

Given the intriguing association between an OTU belonging to *Morganella*, inflammation, and *B4galnt2* genotype, we next set out to more precisely identify and characterize this taxon in our samples, and ultimately test the hypothesis of pathogen-driven selection operating at *B4galnt2* through controlled infection experiments. First, in order to verify the 16S rRNA gene data and resolve the precise location of *Morganella* in the cecum of the wild mice, we designed *fluorescence in situ hybridization* (FISH) probes specific to the 16S rRNA of *Morganella* and stained 46 cecum tissue samples chosen to be representative of *B4galnt2* genotype, inflammation prevalence, and the presence of *Morganella* as detected by 16S rRNA gene analysis. We found 20 animals with positive *Morganella* signal in the cecum, all of which displayed *Morganella* in the lumen (Figure 3a-b, *Suppl. Table 10*), and 12 of which also displayed signal in the intestinal crypts, either associated to the tissue or invading the epithelium (Figure 3a-b, *Suppl. Table 10*). Among the 46 samples, 13 were *Morganella* positive based on 16S rRNA gene sequencing, all of which showed positive signal with FISH, thus confirming the presence of *Morganella* (Figure 3a, *Suppl. Table 10*). In addition, we found seven samples showing positive *Morganella* signals by FISH that were not identified by 16S rRNA gene sequencing. One of these is a healthy heterozygote while the other six are inflamed (two RIIIS/J homozygotes, three heterozygotes and one C57BL/6J homozygote). Overall, only two non-inflamed samples – one RIIIS/J homozygote and one heterozygote – showed detectable levels of *Morganella*, which is consistent with the results of the 16S rRNA gene analysis that found *Morganella* to correlate with inflammation in the set of 169 animals. With regard to *B4galnt2* genotype, these observations are consistent with the larger 16S rRNA gene analysis panel: *Morganella* has the highest prevalence in C57BL/6J homozygotes, while its prevalence in RIIIS/J homozygotes is low, with an intermediate prevalence in heterozygotes. Thus, the FISH results are largely consistent with those obtained by 16S rRNA gene analysis, strengthening *Morganella* as a candidate pathogen linked to *B4galnt2* and inflammation.

Next, we isolated *Morganella* colonies from an inflamed wild mouse homozygous for the C57BL/6J allele class (JJM0912; *Suppl*. Table 2), taking advantage of known antibiotic resistances of members of *Morganella.^29^* This yielded six different isolates, which were subsequently subjected to whole genome sequencing together with three types strains (*Morganella morganii morganii, M. m. sibonii, M. psychrotolerans; Suppl. Table 11*). After sequence assembly and annotation, pairwise genome identities were computed using ANDI,^30^ including two publicly available *M. m. morganii* strain sequences and *Citrobacter rodentium* as an outgroup (*Suppl. Table 11 & 12*). We found four of the six wild isolates to represent the same strain with 100% nucleotide identity (Morg 2, 4, 5, 6), and cluster distinctly from the known *Morganella morganii* subspecies, likely representing a new subspecies of *Morganella morganii* (Figure 3c, *Suppl. Table 12*). The remaining two isolates (Morg 1, 3) are distinct from the previous four isolates and from each other, and cluster closer to the three strains of *Morganella morganii morganii* included in the study, likely representing new strains of this subspecies.

To further characterize these *Morganella* strains, we used the annotations from RAST^31^ and compared the presence/absence of gene functions among strains (*Suppl. Table 13 & 14*). Among the functions specific to Morg 1 and 3 (*Suppl. Table 13*), we find the Phd-Doc toxin-antitoxin system, as well as the colonization factor antigen (CFA/I) fimbrial system. The first system, found only in Morg 3, participates in a toxin-antitoxin (TA) operon involved in a “switch to a quasidormant state” allowing the bacteria to survive external stresses.^32^ The second, present only in Morg 1, was shown to be involved in the pathogenicity of enterotoxigenic *E. coli*,^33^ and could thus be linked to inflammation. The remaining functions are mostly linked to phage activity and metabolism, with no obvious link to *B4galnt2* and/or inflammation.

Next, we examined the functions specific to Morg 2, 4, 5, 6 (*Suppl. Table 14*), which include several pathogenicity-related and metabolic genes that could be related to *B4galnt2*. Each of the four isolates has a copy of two genes related to N-linked glycosylation in bacteria, and two genes related to utilization of N-acetyl glucosamine and N-acetyl galactosamine, which could be involved in using the N-acetyl galactosamine residues transferred by *B4galnt2*. In addition, the four strains each have a copy of two genes involved in flagellar motility, known to participate in a variety of pathogenic phenotypes.^34^ They also harbor a copy of the YkfI TA system, whose function is still poorly understood, but appears to be involved in resistance to oxidative stress and biofilm formation.^35^ Three of the strains harbor a copy of the HigB TA system, shown to reduce the pathogenicity of *Pseudomonas aeruginosa* upon activation.^36^ Importantly, all four strains harbor two copies of the RelE/StbE replicon stabilization toxin, a plasmid gene duo that originates from *Morganella morganii*, but was also found on the enterotoxigenic plasmid P307 of *E. coli* and the chromosome of *Vibrio cholerae* and *Haemophilus influenzae*.^37^ This system is likely to play an important role in pathogenicity, in particular in conjunction with the IncF Conjugal Transfer System involved in the horizontal transfer of plasmids,^38^ and whose 13 detected genes are present in all strains in multiple copies (1 to 4). Finally, all four strains harbor a copy of a gene belonging to the *Listeria* Pathogenicity Island LIPI, which may be involved in the invasion mechanism of *Listeria monocytogenes*.^39^ Thus, overall, the differential gene content between the two classes of *Morganella* taxa isolated in this study suggests that Morg 2, 4, 5, 6 are more likely to possess pathogenic properties that could be linked to *B4galnt2*.

### Experimental infection with M. morganii subspecies confirms B4galnt2 genotype-specific pathology

To experimentally test whether *B4galnt2* genotype is associated with differences in the pathogenicity of *Morganella*, we developed an infection model under standardized laboratory conditions using inbred lab mice differing only according to their *B4galnt2* genotype, similar to the experimental setup we previously used to test the role of *B4galnt2* in *Salmonella* infection.^22^ In order to determine whether the potential effect of *B4galnt2* on infection stems from the lack of gastrointestinal expression, the gain of vascular expression, or a combination of both, we compared infection outcomes in mice with a wild-type copy of the *B4galnt2* gene expressed in the gastrointestinal tract (*B6^+/-^*) to knock-out mice that lack *B4galnt2* expression (*B6^−/−^*).^22^ In combination with the presence or absence of a transgene containing the complete RIIIS/J allele (*RIII^+^* or *RIII^−^*),^22^ it was thus possible to generate the four possible combinations of presence/absence of gastrointestinal and vascular expression on a common C57BL/6J  genetic background. We chose to perform infections with the new *Morganella morganii* subspecies (Morg 2, 4, 5, 6), based on (i) its abundance – the new subspecies represent 4/6 isolated bacteria, and is thus likely to be more abundant in the intestinal microbiota than the two other strains – and (ii) its annotation profile, as it has the potential to be more pathogenic than Morg 1 and 3 and is predicted to have the ability to utilize *B4galnt2*-specific glycan residues.

After an initial antibiotic treatment, mice were gavaged with a suspension of *Morganella*. Mice were kept for 7 days post inoculation (dpi) before sacrifice and organ collection. Colonization was monitored by counting colony forming units (CFUs) in fecal pellets at day four and day seven post inoculation, and in the cecum at the end point (*Suppl. Figure 6*). Successful colonization was reached in all groups, but no significant differences were observed between groups, suggesting that *B4galnt2* does not play a role in the colonization of *Morganella*.

To evaluate whether *Morganella’s* pathogenicity varies according to *B4galnt2* expression patterns, inflammation was measured from cecum histology at the end point (7 dpi), using the same scoring system as for the wild mice. Remarkably, mice lacking gastrointestinal expression (*B6^−/−^*) show significantly lower inflammation in response to *Morganella* infection than mice that do express *B4galnt2* in the gastrointestinal epithelium (*B6^+/-^*), with a median score between 3 and 5, and between 7 and 9, respectively (Figure 4a). This result indicates that the lack of *B4galnt2* expression in the gastrointestinal tract effectively ameliorates inflammation caused by *Morganella*. In addition, among mice lacking gastrointestinal expression (*B6^−/−^*), those that possess the RIIIS/J transgene (*RIII^+^*) have significantly lower inflammation scores than those that do not express *B4galnt2* in the vascular endothelium (*RIII^−^*) (Figure 4a). This suggests that vascular *B4galnt2* expression further improves the protection provided by the lack of gastrointestinal expression. As conducted for the wild-caught mice, we independently validated our inflammation scoring by measuring the inflammatory cytokines *Il1b, Ifng*, and *Mcp1* via qPCR. All three markers display a significant positive correlation to the inflammation score based on histological scoring (*Il1b* rho = 0.656, p < .001; *Ifng* rho = 0.450, p = .013; *Mcp1* rho = 0.507, p = .003). Further, *Il1b* and *Mcp1* also display significant differences according to *B4galnt2* genotype, whereby *Il1b* in particular appears to mirror the pattern observed based on histological scoring (*Suppl. Figure 7*).

To further characterize the link between *Morganella* and inflammation in this model, we performed FISH as described for the wild mice. This reveals adherent and invasive *Morganella* to be present in all genotypes (Figure 4d). Moreover, a trend of association between the inflammation score and the number of adherent and invasive *Morganella* is observed only among *B6^+/-^*/*RIII^−^* animals (Spearman p = 0.0663; rho = −0.75 for adherent and rho = 0.75 for invasive), suggesting a link between *Morganella* and inflammation in a *B4galnt2* genotype-dependent manner (Figure 4b-c). Thus, remarkably, the outcome of controlled infection experiments in the lab highly mimic the relative differences according to the *B4galnt2* genotype observed in the wild.

## Discussion

In this study, we experimentally tested the hypothesis that variation at the *B4galnt2* gene mediates susceptibility to intestinal pathogens in wild house mouse populations. This work was motivated by striking signatures of selection^17–19^ combined with a strong detrimental bleeding phenotype associated with allelic variation at this locus, which is present in both laboratory- and wild mouse populations.^17,23^ Evolutionary trade-offs are often assumed in cases where disease phenotypes intersect with signatures of selection, and represent a general explanation for the maintenance of diseases associated with genetic variation in natural populations. However, the nature of such trade-offs is often unclear and rarely experimentally tested. Here, we employed a unique combination of culture-independent analysis of bacterial communities and histopathology in a large number of wild mouse gut samples and identified a *M. morganii* strain as a novel enteric pathogen in mice, to which differences in susceptibility are mediated by variation at *B4galnt2*.

In addition to providing a useful approach to identify and monitor sources of disease in wild animals, our results add to the expanding role of *B4galnt2* in both infectious- and other classes of disease (reviewed in^21,40^) and provide important experimental insight into interpreting signatures of selection at this locus. *B4galnt2* is long known to underlie the synthesis of the Sd^a^ antigen, and in addition to modifying levels of von Willebrand factor in mice, plays a role in mediating human colon cancer metastasis, the lytic function of cytotoxic T lymphocytes in mice, and prevents muscular dystrophy in murine models.^40^ Our previous work reveals gastrointestinal expression of *B4galnt2* to influence both resident bacteria^24^ and susceptibility to experimental *Salmonella* infection.^22^ Finally, other recent studies demonstrate *B4GALNT2* can inhibit both influenza A and several avian influenza strains in human cell lines.^41,42^ The diversity of tissues and bodily secretions in which the Sd^a^ antigen is present (e.g. saliva, intestinal epithelium, milk, and urine^43^), along with the likely high number of protein and lipid targets of B4GALNT2 glycosylation, could represent a general explanation for this range of phenotypes and propensity for pleiotropic effects.

In principle, the fitness effects surrounding *B4galnt2* genotype could be associated with a loss of intestinal expression and/or a gain of vascular expression. In the intestinal mucosa, the major secreted protein comprising the mucus layer, MUCIN 2 (MUC2), appears to be one important target of glycosylation by B4GALNT2,^22,44^ which could play a role in the adherence and/or invasion by *Morganella*. Based on our controlled laboratory experiments, the largest impact on inflammation score is indeed the presence/absence of intestinal expression (Figure 4a). However, among the mice lacking intestinal expression, we also observe a significant reduction in inflammation score in mice *with* vascular expression compared to those *lacking* vascular expression (Figure 4a). Of note, our previous study of *Salmonella* infection displayed a very similar relative pattern, although only intestinal expression was significant for inflammation score and vascular expression for colonization levels.^22^ Despite the significant, albeit smaller effects of vascular expression in both of the aforementioned models, we do not expect a direct role of a bleeding phenotype per se. In neither model is the intestinal inflammation severe enough to cause intestinal bleeding. Further, the prolonged bleeding time observed in mice with *B4galnt2* vascular expression is an indirect effect resulting from aberrant B4galnt2-glycosylation of von Willebrand factor (VWF), which leads to accelerated VWF clearance and prolonged bleeding upon injury.^20^ Interestingly, recent advances in our understanding of the coagulation system indicate that it is also generally a partner in innate immunity and host defenses,^45^ including a direct role of VWF itself in regulating macrophage function.^46^ Thus, while we cannot rule out a possible role of differences in VWF or other targets of B4galnt2 glycosylation in blood vessels contributing to our observations, bleeding time per se likely represents a general fitness cost in nature, rather than playing a direct role in the phenotypes observed in wild and laboratory mice in this study.

In natural populations of *M. m. domesticus*, the effects of the C57BL/6J and RIIIS/J allele classes are co-dominant regarding the presence of expression, i.e. heterozygotes display expression in both tissues, whereas homozygotes display only intestinal- or vascular expression, respectively.^17^ Importantly, our previous theoretical analysis of scenarios that could lead to long-term maintenance of the two allele classes found that (i) pathogen-driven selection can produce the *B4galnt2* genotype frequencies observed in nature when both heterozygotes and RIIIS/J homozygotes are protected against infection and the fitness cost of bleeding is approximately half that of infection, and (ii) a dominant, protective function of the RIIIS/J allele appears to be more important for long-term maintenance than a loss of intestinal expression.^19^ Intriguingly, our observations regarding *Morganella* in the wild appear to fit these predictions, as mainly a preponderance of inflamed C57BL/6J homozygotes is present among individuals for which *Morganella* is detected (Figure 2). Taking both the controlled laboratory experiments and observational data in the wild into account, a contribution of losing intestinal expression can, however, not be easily ruled out. Given the variety of differences in susceptibility to pathogens for blood group-related genes in general and that genotype at a given locus can have opposing fitness effects based on the individual pathogen at hand,^21^ it is likely that the actual selective regime in nature is complex and involves numerous pathogens and could potentially also include VWF.

Nonetheless, our results do also provide potentially important new insight into the infection biology of *Morganella spp*, which includes important opportunistic pathogens that can, e.g., lead to sepsis, abscesses, chorioamnionitis, cellulitis, and urinary tract infections, the latter of which can result in “purple urine bag syndrome”.^29^ Interestingly, both a urinary tract mucin and the abundant urinary Tamm-Horsfall glycoprotein (a.k.a. Uromodulin) are known to carry the Sd^a^ antigen,^40^ thus, it can be reasonably speculated that *B4GALNT2* may contribute to urinary tract infections involving *Morganella*. Given (i) the intrinsic multi-drug resistance of *M. morganii*,^47^ (ii) the recent observation of sewage isolates of *M. morganii* harboring a resistance gene to the clinically important, last-resort antibiotic colistin,^48^ and (iii) the ability of *Morganella* to transfer antibiotic resistance to other pathogens,^49^ future mechanistic work surrounding the potential interactions between *Morganella* and B4GALNT2-derived glycan structures is clearly warranted.

In summary, the pathometagenomic approach taken here leads to an important confirmation of predictions made by population genetic- and disease-associated phenotypic data surrounding *B4galnt2* and identifies wild mice as potentially important environmental reservoirs of a clinically important opportunistic pathogen. Interestingly, these results suggest that the risk of zoonotic transfer may depend on population genetic aspects of resistance/susceptibility alleles in a given reservoir species. Thus, it is conceivable that future work could expand the approach outlined here to the level of the entire hologenome, which could be more broadly applied as a means to detect potential emerging pathogens.

## Materials and methods

### Field work and sampling

Two hundred and seventeen wild house mice were caught around the southwestern French town of Espelette, during a five-week field work in September 2013 (first described in^26^). Animals were live-trapped in 34 randomly chosen farms and brought back to a common location where they were euthanized with CO_2_ and dissected on site. The presence of farm animals and use of poison was recorded for each farm (*Suppl*. Table 1). The pairwise distance between farms was calculated from their GPS coordinates with the “haversine” formula (*Suppl. Table 3*). Families were defined as groups of farms within which all farms are at most 2 km apart from each other, as suggested in;^27^ “super families” were defined as groups of farms within which all farms are at most 3.5 km apart from each other (*Suppl*. Tables 1 *& 3*, Figure 1a). For each mouse, body and tail length, weight and gender were recorded. The body mass index (BMI) was calculated as BMI = W/L,^2^ where W is the weight in kilograms, and L is the body length in meters (*Suppl*. Table 2). For the purposes of microbial analysis and histology, we chose the cecum, as it is the location in the gastrointestinal tract that harbors the most bacteria in mice, and because we had prior knowledge that *B4galnt2* genotype can influence both microbiota and susceptibility to *Enterobacteriaceae* such as *Salmonella* Typhimurium^22^ or *Citrobacter rodentium*^50^ at this location. Further, little to no ileal inflammation is observed for either of the two above mentioned pathogens. The cecum was accordingly sampled in four pieces transversally (*Suppl. Figure 8*) and used for microbial analysis and histology. The tip of the cecum, thereafter referred as *Cecum4* was stored in *AllProtect* (Qiagen) at +4°C; *Cecum3* was stored in 10% formalin at +4°C; *Cecum2* was stored in pre-reduced brain-heart infusion (BHI) with 20% glycerol at −80°C; *Cecum1* was store in *RNAlater* at +4°C for 24 h, before removing the stabilizing solution and long-term storage at −20°C. A piece of the right ear was stored at −20°C and used for genotyping.

### Nucleic acid and protein extraction

Right ear samples (for mitochondrial sequencing, microsatellite typing, *B4galnt2* genotyping) were extracted using the DNeasy Blood & Tissue Kit (Qiagen) according to manufacturer’s instructions. *Cecum4* samples were washed in 1xPBS and placed in 600 µL of RLT buffer (Qiagen) containing 24 µL of 0.5 M TCEP (Tris(2-carboxyethyl)phosphine hydrochloride, Sigma Aldrich) in a Lysing Matrix E tube (MPBio). These samples were homogenized 3x15s at 6500rpm in a Precellys 24 (Bertin Instruments). The lysates were passed through QIAshredder spin columns (Qiagen), and extracted with the AllPrep DNA/RNA/Protein kit (Qiagen) according to manufacturer’s instructions. After elution, samples were quantified on a NanoDrop (ThermoScientific). cDNA was synthesized using the High-Capacity cDNA Reverse Transcription Kit (Applied Biosystems).

### B4galnt2 genotyping and mitochondrial D-loop sequencing

A diagnostic PCR fragment was sequenced as a means to identify the presence of alternative *B4galnt2* allele classes as previously described.^18^ An 885 bp portion of the mitochondrial D-loop was sequenced as previously described.^25,26,51^ Sequencing was performed on an ABI 3730 automated sequencer (Applied Biosystems) and sequences were edited in GENEIOUS 7.0 (Biomatters Ltd) and transferred to MEGA 5^52^ for alignment to reference sequences and previous data sets. For *B4galnt2*, reference sequences were taken from.^17^ For the mitochondrial D-loop, reference sequences for *Mus musculus domesticus, Mus spretus*, and *Mus spicilegus* were used (GenBank Accessions: AM182648, U47539, and U47536, respectively), as well as sequences from.^25^ A NeighbourNet network was constructed using the SplitsTree package (v.4.10)^53^ to determine mitochondrial haplogroups as described in^25,26,54^ (*Suppl*. Figure 1).

### Microsatellite typing and analysis

Eighteen neutral autosomal loci described in^55,56^ were typed in Geneious (v.7.0) and analyzed using the software STRUCTURE (2.3.4)^57–59^ as described in^26^ (*Suppl. Table 4*). Individuals that showed >80% ancestry from one cluster were considered reliably assigned to that cluster. In order to assign population groups to individuals with admixed ancestry, cluster membership from STRUCTURE (*Suppl. Table 5*) was used to calculate Euclidean distances between individuals using the function “vegdist” from package vegan^60^ in R and build a neighbor-joining tree (*Suppl*. Figure 2). Pairwise relatedness between animals was calculated in KINGROUP^61^ using the Kinship method (*Suppl*. Figure 3, *Suppl. Table 6*).

Twelve microsatellites spanning the *B4galnt2* gene region described in^18^ were typed in Geneious (v.7.0) and phased according to *B4galnt2* haplotypes using PHASE 2.1^62^ (*Suppl. Table 7*). Ten thousand iterations were performed, with a thinning interval of 100 and a burn-in of 10,000. Consistency between runs and selection of the best run was conducted using the “_freqs” and “_monitor” output files, respectively, as suggested by the developers. GenoDive 2.0^63^ was used to calculate expected heterozygosity.^64,65^

### Histopathology

*Cecum3* samples (*Suppl. Figure 8*) were fixed in 10% formalin, dehydrated with ethanol and embedded in paraffin. Paraffin sections were stained with hematoxylin–eosin (H&E) according to standard laboratory procedures. Intestinal inflammation was assessed through the severity of epithelial desquamation (0–3), necrotic epithelial cells in the lumen (0–3), and infiltration of polymorphonuclear (PMN) leukocytes in the mucosa (0–3) and the submucosa (0–3), for a total score ranging from 0 to 12. The scoring was performed by two independent pathologists, and the mean of the total scores was used for further analysis (*Suppl. Table 8*). All scoring was done blinded to the mouse genotype. Inflammation prevalence was inferred from the scores, whereby mice with a total score of zero were identified as “healthy”, and mice with a score higher than zero were identified as “inflamed”.

### Quantitative PCR

qPCR was performed using fluorescently labeled assays from Integrated DNA Technologies (IDT) for wild-caught mice, and SYBR Green assay (Roche) for lab mice and *Il1b*.

IDT assays are as follow:

- *Hprt1*: Mm.PT.58.29815602

- *Ifng*: Mm.PT.58.41152792

- *Mcp1*; Mm.PT.58.42151692

SYBR Green primers;

- *Hprt1*: fw-AGTGTTGGATACAGGCCAGAC, rev-CGTGATTCAAATCCCTGAAGT

- *Ifng*: fw-TCAAGTGGCATAGATGTGGAAGAA, rev-TGGCTCTGCAGGATTTTCATG

- *Mcp1*: fw-CCTGCTGTTCACAGTTGCC, rev-ATTGGGATCATCTTGCTGGT

- *Il1b*: fw-TGTGAAATGCCACCTTTTGA, rev-GGTCAAAGGTTTGGAAGCAG

PCR mix for IDT assays was prepared with the Agilent Brilliant Probe Multiplex Master Mix (Cat No 600553) as follow: 0.5ul of 20x target gene assay, 0.2ul of 20x housekeeping gene assay, 5ul of Agilent master mix, qsp to 8ul with RNase free water, and 2ul of sample cDNA. SYBR Green assay was performed with SYBR-Green Mastermix (Roche) and gene-specific primers. 2–3 technical replicates per samples were processed on a PikoReal 96 Real-Time PCR System, with the following program: 95°C 10 min; 95°C 15 sec, 60°C 60 sec*; 60°C 30 sec; 50 cycles; melt ramp 60°C-95°C*, 4°C 10 sec; *data acquisition. Samples with standard deviation between technical replicates above 0.9 were excluded. Relative expression was calculated from Ct values using the 2^−ΔΔCT^ method, using *Hprt1* as a housekeeping gene and the RIIIS/J or B6^−/−^ RIII^+^ group average as a normalizing factor for wild-caught mice and lab mice, respectively.

### 16S rRNA gene sequencing & processing

The V1-V2 region of the 16S rRNA gene was amplified with a dual indexing approach using bacterial universal primers 27 F and 338 R on a MiSeq (Illumina) as described in.^26^ DNA and cDNA template from *Cecum4* (*Suppl. Figure 8*) were used, along with their respective negative extraction controls (*Suppl. Table 15*).

Raw sequences were quality filtered and trimmed using CUTADAPT,^66^ forward and reverse reads were merged, filtered, dereplicated, and denoised with USEARCH v11.0.667.^67,68^ Sequences were further processed with MOTHUR v.1.44.3.^69^ Briefly, sequences were aligned to the Silva 132 reference database,^70^ classified with the RDP trainset 16 databases,^71^ and clustered into 97% identity operational taxonomic units (OTUs). Subsampling was performed by randomly sampling 10,000 unique sequences with replacement, using their abundances as probabilities. The rounded median from 1,000 iterations was used for further analysis. Complete scripts for processing of sequences are available on figshare (see Data availability). Some extraction negative controls yielded positive amplification and sequencing, thus, SourceTracker^72^ was used to identify potentially contaminated samples. Samples with more than 10% of contamination source coming from the same extraction batch or more than 40% contamination from all sources were excluded from the analysis.

### 16S rRNA gene analysis

OTU count tables were imported in R for further analysis. Only samples that yielded successful sequencing for both DNA and cDNA dataset were considered for further analysis. A combined prevalence table was constructed by considering an OTU present when detected in either one or both data sets. Activity was defined as the ratio between abundance in cDNA relative to abundance in DNA, plus one. The relative activity was obtained by normalizing the activity by sample.

The vegan package^60^ was used for beta diversity measures. Bray-Curtis distances were calculated with vegdist from the abundance and activity dataset, while Jaccard distances were calculated from the prevalence dataset. Principal coordinate analysis (PCoA) was performed with cmdscale, and the influence of environmental and host factors on the microbiota was tested by permanova (adonis) with 10,000 permutations. Explanatory variables were first tested alone (*Suppl. Table 16*), then added in intermediate models (*Suppl. Table 17*) and finally added to a complete model (Table 1).

For the identification of candidate pathogens, a core community was defined by selecting the union of the 99% most abundant OTUs per sample and filtering out OTUs present in less than five samples, and whose maximum relative abundance was lower than 0.1%, yielding the final data set to comprise 727 OTUs. The influence of eight variables (listed below) on each OTUs was assessed through a linear model approach adapted from a previous analysis of this mouse population focusing on skin microbiota.^26^ Abundance, activity, and prevalence of each OTU were used as variables of interest in independent models that used the following parameters as explanatory variables in the following order: sequencing library, super family, population, haplogroup, weight, *B4galnt2* genotype, inflammation score, and the interaction between *B4galnt2* genotype and inflammation score. These parameters were chosen to identify OTUs associated to *B4galnt2* genotype and inflammation, while accounting for parameters found to influence the microbiota composition in the ecological analysis. Candidates were selected by requiring the OTUs to be significantly associated (p < .05) with both *B4galnt2* genotype and inflammation score in a model that also reached overall significance.

### Candidate pathogen isolation

In order to maximize the chances of recovery, samples were selected based on the abundance of *Morganella* relative to other *Enterobacteriaceae*, as observed in the 16S rRNA gene profiling. Selected *Cecum2* samples (*Suppl. Figure 8*) were diluted 1:10 in PBS/40% glycerol and homogenized. 5 µl of homogenate was inoculated in 2.5 ml of LB medium containing oxacillin (20 µg/ml), vancomycin (20 µg/ml), erythromycin (20 µg/ml), and cefaclor (20 µg/ml), or only cefaclor (20 µg/ml). The liquid cultures were incubated overnight at 37°C with shaking. Serial dilutions were plated on MacConkey, LB, and Columbia Blood agar. Single colonies were picked and spread on MacConkey and Columbia Blood agar. Fresh colonies were identified using MALDI-TOF, revealing six strains belonging to *Morganella*. The strains displayed two different colony morphologies: rough, frayed, and round colonies stemming from the first selection with four antibiotics (isolates 2, 4, 5, 6); lighter, round, and smooth colonies stemming from the second selection with cefaclor only (isolates 1, 3).

### Candidate pathogens whole genome sequencing

Genomic DNA of *Morganella* isolates was extracted using the DNeasy UltraClean Microbial Kit (Qiagen) following the manufacturer’s instructions. Isolates were prepared with the Nextera XT DNA Library Prep kit (Illumina). Libraries were sequenced with the V2 kit (2x250 bp) on a MiSeq (Illumina). Demultiplexing was performed with Casava (Illumina) using the “eamss” algorithm. Forward and reverse reads were merged using usearch v8.1.1861,^73^ with the following options: truncate the read at the first base with quality 5 or below; the truncated read should be ≥100 bp long, and the overlap between forward and reverse should be ≥100 bp long. The quality of the libraries was evaluated with FastQC. Genome assembly was performed in GENEIOUS 7.0 (Biomatters Ltd), using the Geneious assembler with increasing sensitivity: first, the “Medium-Low Sensitivity” was used, then the “Medium Sensitivity”, and finally the “High Sensitivity”. For the second and third assembly steps, contigs from the previous assembly were kept unchanged, and assembled with reads that could not be assembled on the previous step. Low coverage regions (<10 reads) and variants/SNPs (minimum coverage 10, minimum variant frequencies 0.1) were detected using the Geneious tool and inspected manually. Low coverage regions were trimmed out, and contigs formed of less than 100 reads, with an average coverage lower than 10 were filtered out. Consensus sequences were exported as fasta files using the majority option and annotated by online RAST,^31^ using the “classic RAST” annotation scheme, the “RAST” gene caller, and the “fix error” and “backfill gaps” options. The RAST comparison tool was used to evaluate the differences between the strains.

Evolutionary distances between the candidate pathogens and reference genomes was calculated with ANDI^30^ with Jukes-Cantor model, 10,000 iterations, and excluding plasmid sequences. Distance matrices were imported in R via the function *as.dist* from the *VEGAN* package,^60^ and used to build a neighbor joining tree for each iteration using the function NJ from the *PHANGORN* package.^74^ The package *APE*^75^ was used to build a consensus tree from the 10,000 iterations.

### Candidate pathogen detection by fluorescence in situ hybridization (FISH)

Formalin-fixed and paraffin-embedded *Cecum3* (*Suppl. Figure 8*) tissue sections (5 μm) were deparaffinized and rehydrated. Bacteria were detected with probes complementary to a conserved region of the 16S rRNA gene. *Morganella* were visualized with a combination of two probes:

- MORG580a 5’–[Cy3]CTGACTCAATCAACCGCCTGCG-3´;

- MORG580b 5’–[Cy3]CTGACTCAGTCAACCGCCTGCG-3’).

The probes (5 µM) were incubated in warm hybridization buffer (0.9 M NaCl, 0.1 M Tris pH 7.2, 0.1% SDS) overnight in a humid chamber at 37°C in the dark. The slides were washed with warm hybridization buffer followed by wash buffer (0.9 M NaCl, 0.1 M Tris pH 7.2) for 15 min each with gentle shaking. Tissue sections were mounted using Prolong Gold mounting medium containing DAPI. Images were obtained on a Zeiss Apotome.2 microscope using AxioVision 4.9.1 software (Zeiss).

### Animal experiment

Heterozygous *B4galnt2* knock-out allele (*B6^+/-^*) and RIIIS/J-*B4galnt2* BCA transgenic (*RIII^+^*) which exhibit the Mvwf1 phenotype were re-derived at Institute for Laboratory Animal Science, Hannover Medical School. Heterozygous breeding pairs produced litters of mixed genotype (*B6^−/−^RIII^−^; B6^−/−^RIII^+^; B6^+/-^RIII^+^; B6^+/-^RIII^−^*). Mice were housed together under specific pathogen-free conditions in individually ventilated cages (IVC). Standard chow and water were provided ad libitum. Experiments were conducted in the animal facilities of the Hannover Medical School in Germany. Animal experiments were conducted in direct accordance with the German Animal Protection Law consistent with the ethical requirements and approval of the Animal Care Committee of the Niedersächsisches Landesamt für Verbraucherschutz und Lebensmittelsicherheit (protocol # 18/2747).

Ampicillin (0.5 g/L) was administered via drinking water from day 6 before infection until day 7 after infection. For infection, mice were orally gavaged with 10^9^ *Morganella morganii* which were grown in LB medium containing oxacillin (20 µg/ml), vancomycin (20 µg/ml), erythromycin (20 µg/ml), and cefaclor (20 µg/ml) for overnight at 37°C with shaking. Body weight was monitored 2–3 times weekly. Mice were sacrificed at day 7 after infection. To enumerate bacteria, cecum content was harvested, homogenized, serially diluted, and plated on LB agar containing oxacillin (20 µg/ml), vancomycin (20 µg/ml), erythromycin (20 µg/ml), and cefaclor (20 µg/ml).

Cecum tissues were fixed in 10% formalin, dehydrated with ethanol, and embedded in paraffin. Paraffin sections were stained with hematoxylin–eosin (H&E) and histopathological changes were assessed as described above (*Suppl. Table 18*). FISH was performed as described above, and the number of adherent and invasive *Morganella* was counted in 10–14 fields of view (FOV) per sample (*Suppl. Table 19*). The average counts across FOV were used for statistical analysis.

RNA was extracted from mouse cecal tissue using the High Pure RNA Tissue Kit (Roche). RNA was reverse transcribed into cDNA using the cDNA Synthesis Kit (Roche) according to the manufacturer’s instructions.

### Statistics

Correlations between continuous variables were tested using Spearman correlations; associations between continuous and categorical variables were evaluated with Kruskal-Wallis or Wilcoxon tests, and comparisons between categorical variables were evaluated with χ^2^ tests. All analyses were performed in *R*.^76^ Correction for multiple testing was done using the false-discovery rate (FDR)^77^ method where applicable.

### Data and code availability

All data on wild and laboratory mice are available as tables in the article or in Suppl. Tables. D-loop sequences are available in GeneBank with accession numbers MN027281-MN027496 in the PopSet 1916749452^26^. Sequences and genomes are available in GeneBank, under the bioproject PRJNA707395: accession numbers SRR14060992-SRR14061481 for 16S rRNA gene sequences, biosamples SAMN25335499-SAMN25335507 for WGS sequences and draft genomes. Data and scripts for 16S rRNA gene analysis are available on figshare: 10.6084/m9.figshare.14160980 for input data, 10.6084/m9.figshare.14160323 for processing scripts, and 10.6084/m9.figshare.14160971 for analysis scripts and datasets. RAST-annotated genomes are available on figshare (10.6084/m9.figshare.19067720).

## Supplementary Material

Supplemental Material
