# Supplementary material for: Pathometagenomics reveals susceptibility to intestinal infection by Morganella to be mediated by the blood group-related B4galnt2 gene in wild mice
Source: Gut Microbes. 2023 Jan 22;15(1):2164448. doi: 10.1080/19490976.2022.2164448 (PMC9872957; doi:10.1080/19490976.2022.2164448)
Supplement: Supplemental Material [file KGMI_A_2164448_SM1167.zip › Vallier_et_al_2023_Supplementary_Figures.pdf]

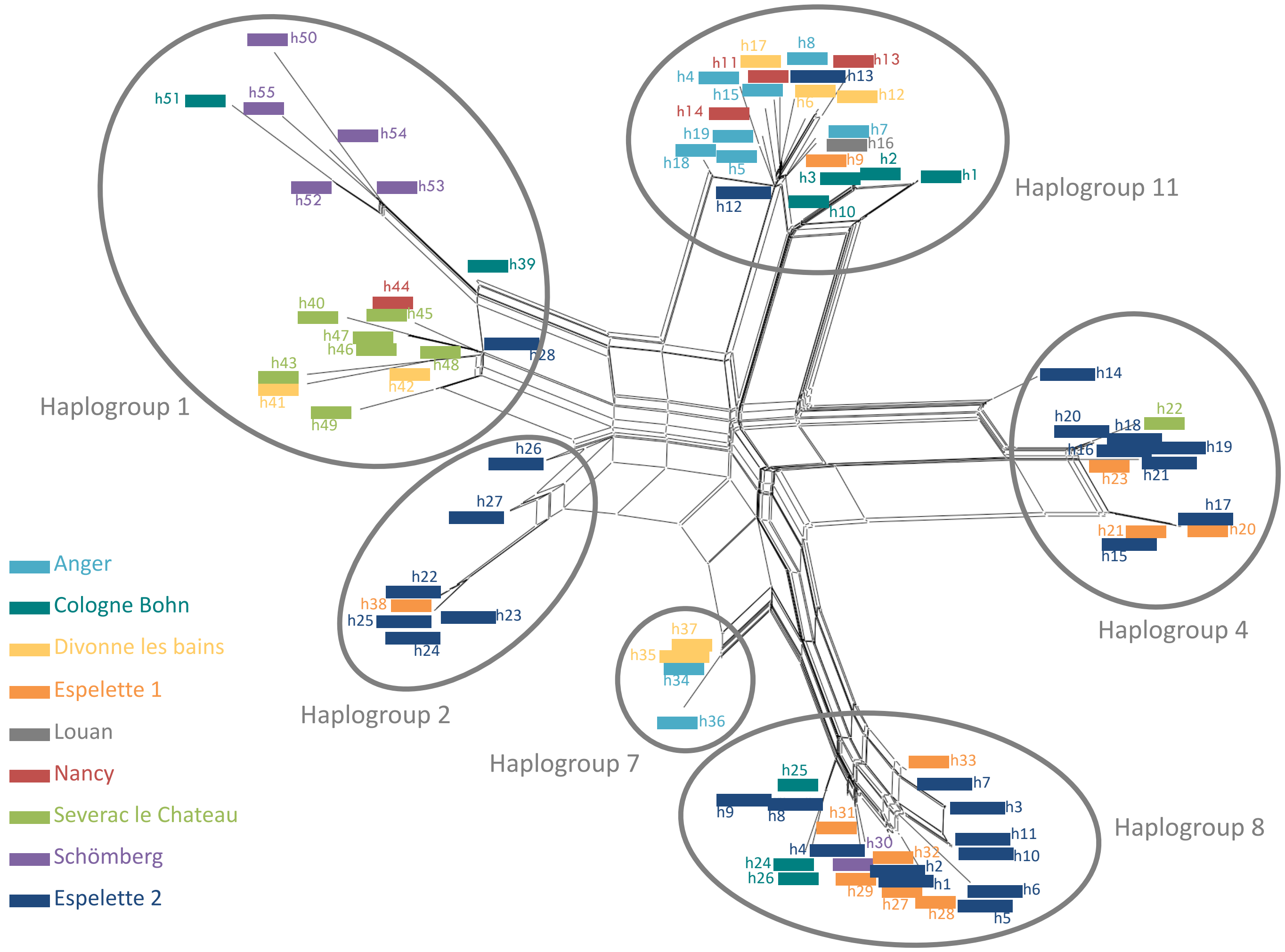


**Supplementary Figure 1: NeighbourNet network of 87 unique mitochondrial D-loop sequences.** 55 unique haplotypes from eight previously sampled locations were included as reference (Espelette 1)[25] together with 28 unique haplotypes from this study (Espelette 2). The population is composed of five haplotype clusters (haplogroups, numbered according to[25,51]), all observed in the previous collection.

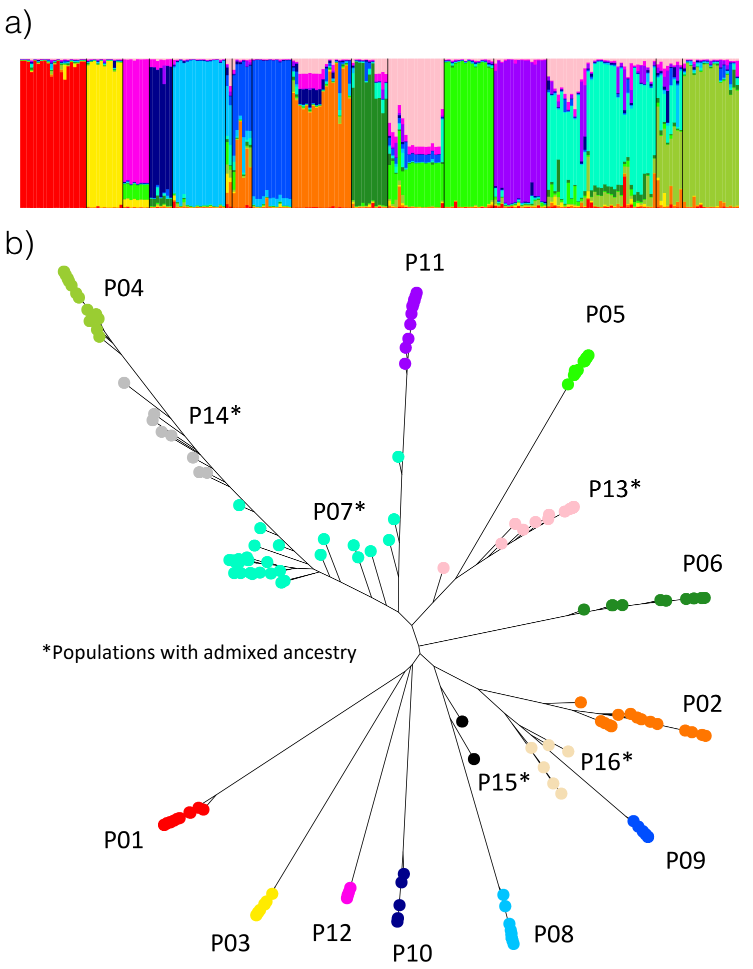


**Supplementary Figure 2: Population structure of 217 mice caught in southwest France. a)** Genetic clusters from STRUCTURE with k=13. Vertical bars show ancestry for individual mice. **b)** Neighbor-joining tree from Euclidean distance between mice, based on the proportion of membership to each of the 13 genetic clusters, reveals 16 distinct mouse populations.

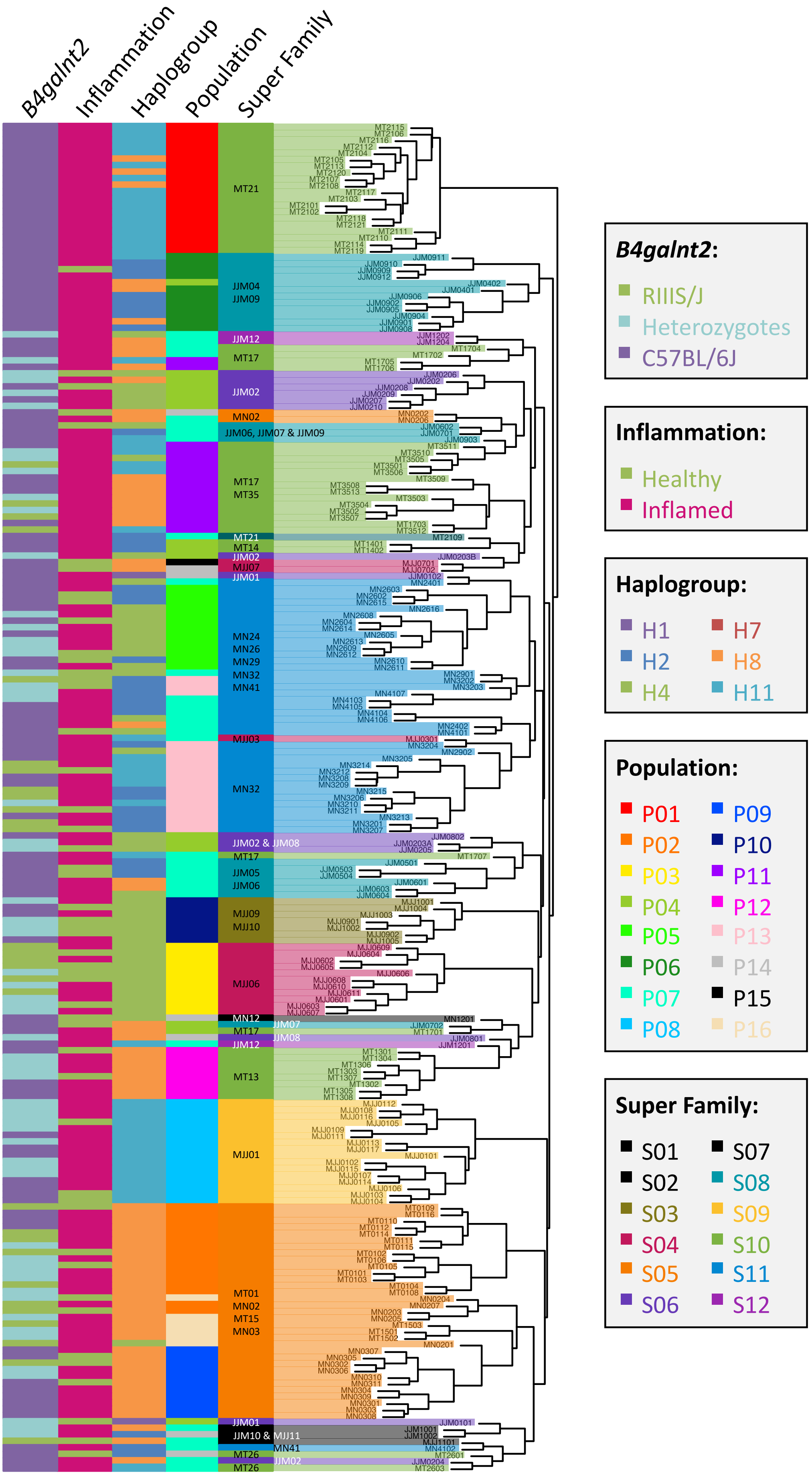


**Supplementary Figure 3: Relatedness of 217 mice caught in southwest France.** Hierarchical clustering based on the kinship matrix of relatedness, calculated from 18 neutral microsatellite markers. Columns show *B4galnt2* genotype, cecum inflammation, mitochondrial D-loop haplogroup, population and super family as colored bars.

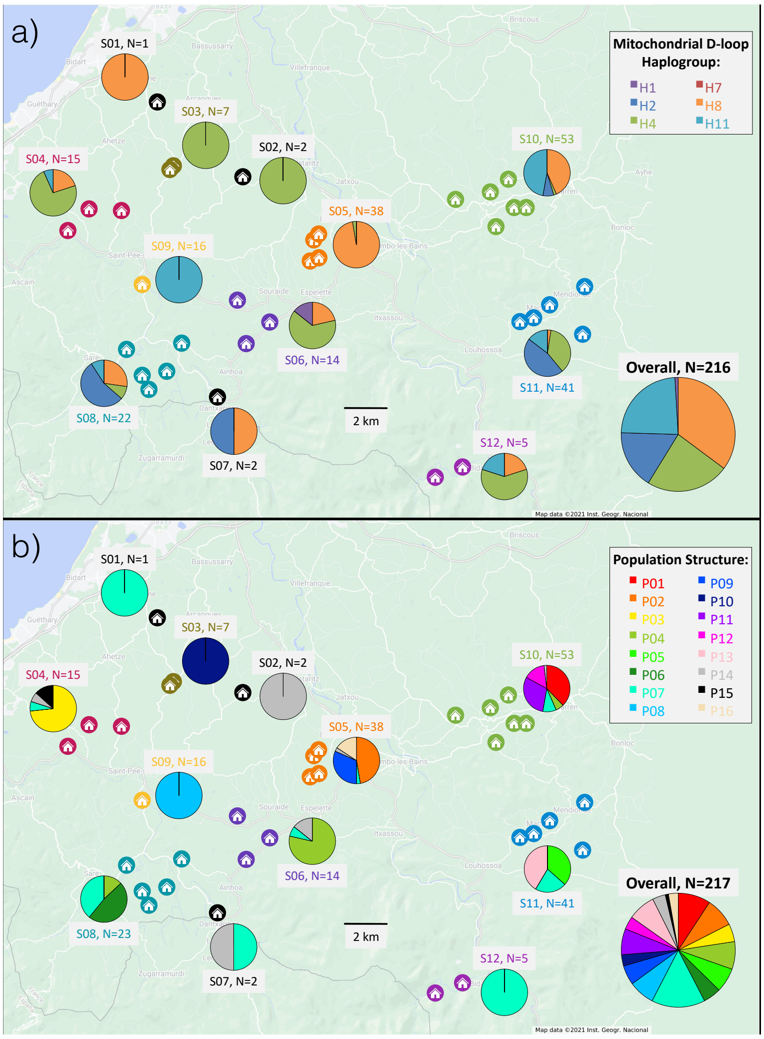


**Supplementary Figure 4: Mitochondrial haplotypes and population structure.** Mitochondrial D-loop haplogroup **(a)** and population **(b)** distribution at 12 super families sampled in southwest France, and overall distribution. For each super family, the identifier (S##) and sample size are shown.

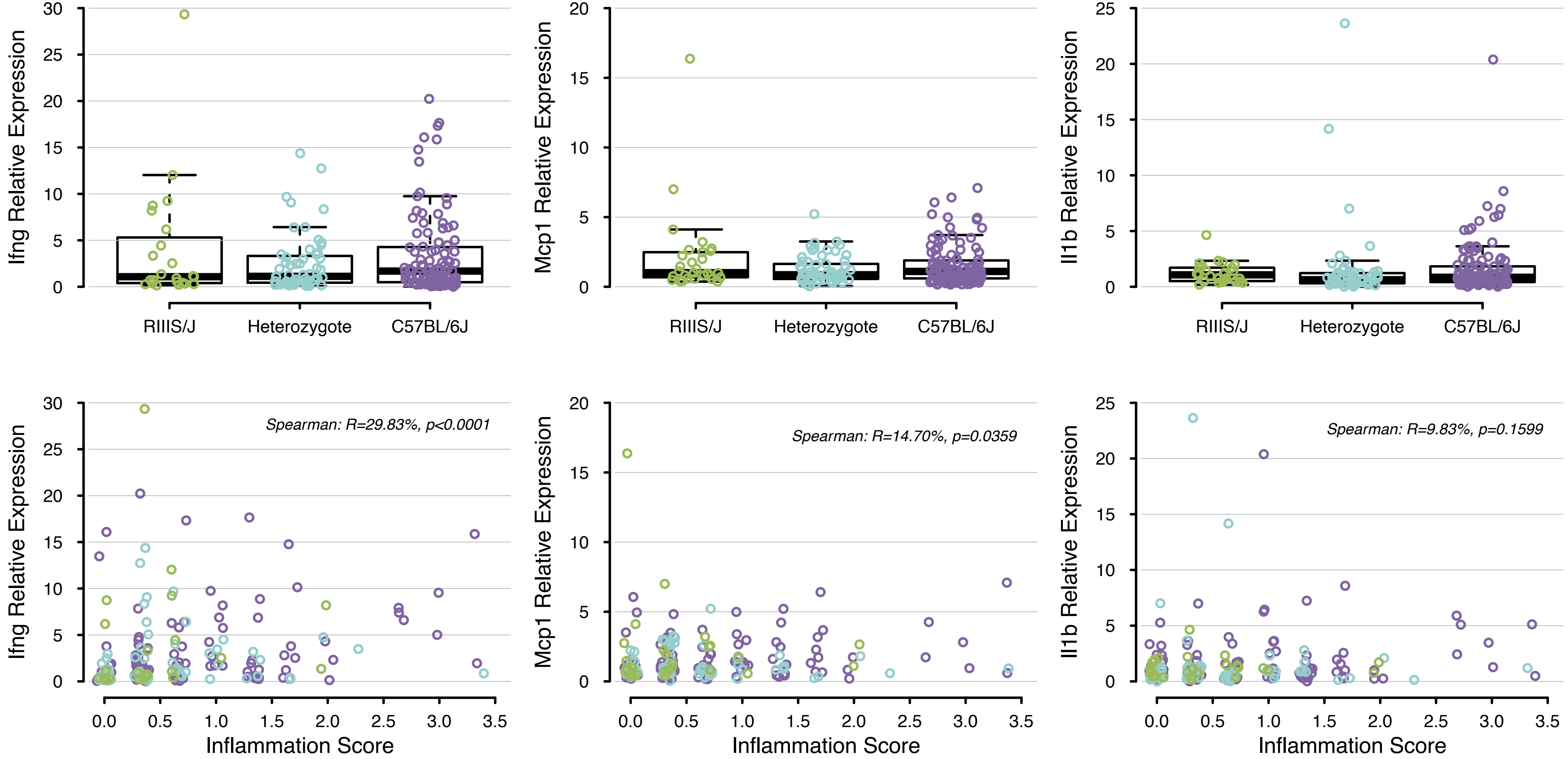


**Supplementary Figure 5: Relative expression of immunity genes in wild-caught mice.** Relative expression of IFNg (left), MCP1 (center) and Il1b (right) according to *B4galnt2* genotype (top) and histology-based inflammation score (bottom) in wild-caught mice. Relative expression was calculated through the delta delta Ct method by normalization to the house-keeping gene HPRT1, and the mean detla Ct of the RIIIS/J group. Colors in the bottom panels represent *B4galnt2* genotype as displayed in the top panels. Comparisons between genotype categories in the top panels were made using pairwise Wilcoxon tests with “FDR” correction for multiple testing, although no comparison is significant.

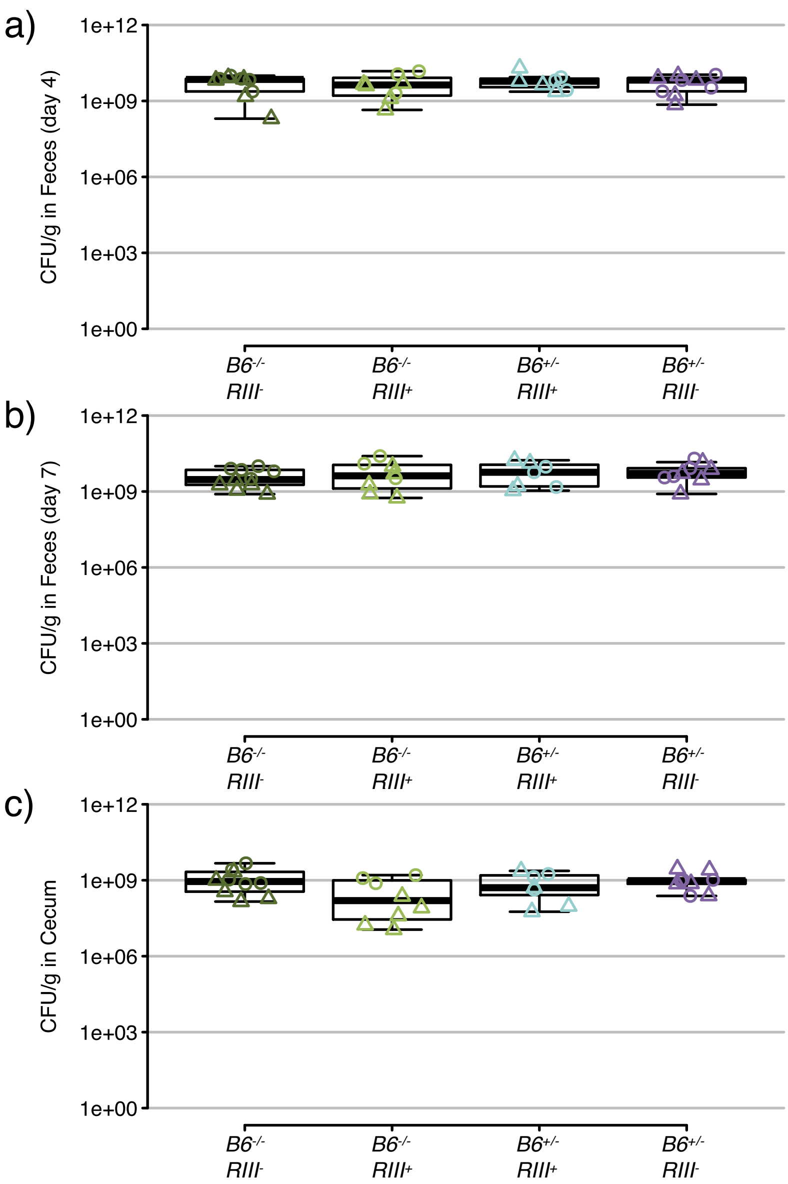


**Supplementary Figure 6: *Morganella* successfully colonizes laboratory mice.** *Morganella* CFU counts of C57BL/6J mice experimentally infected with *Morganella* according to *B4galnt2* genotype/expression category. **a)** Fecal CFUs at day 4 post infection. **b)** Fecal CFUs at day 7 post infection. **c)** Cecal CFUs at endpoint. Comparisons between genotype categories were made using pairwise Wilcoxon tests with "FDR" correction for multiple testing, although no comparison is significant.

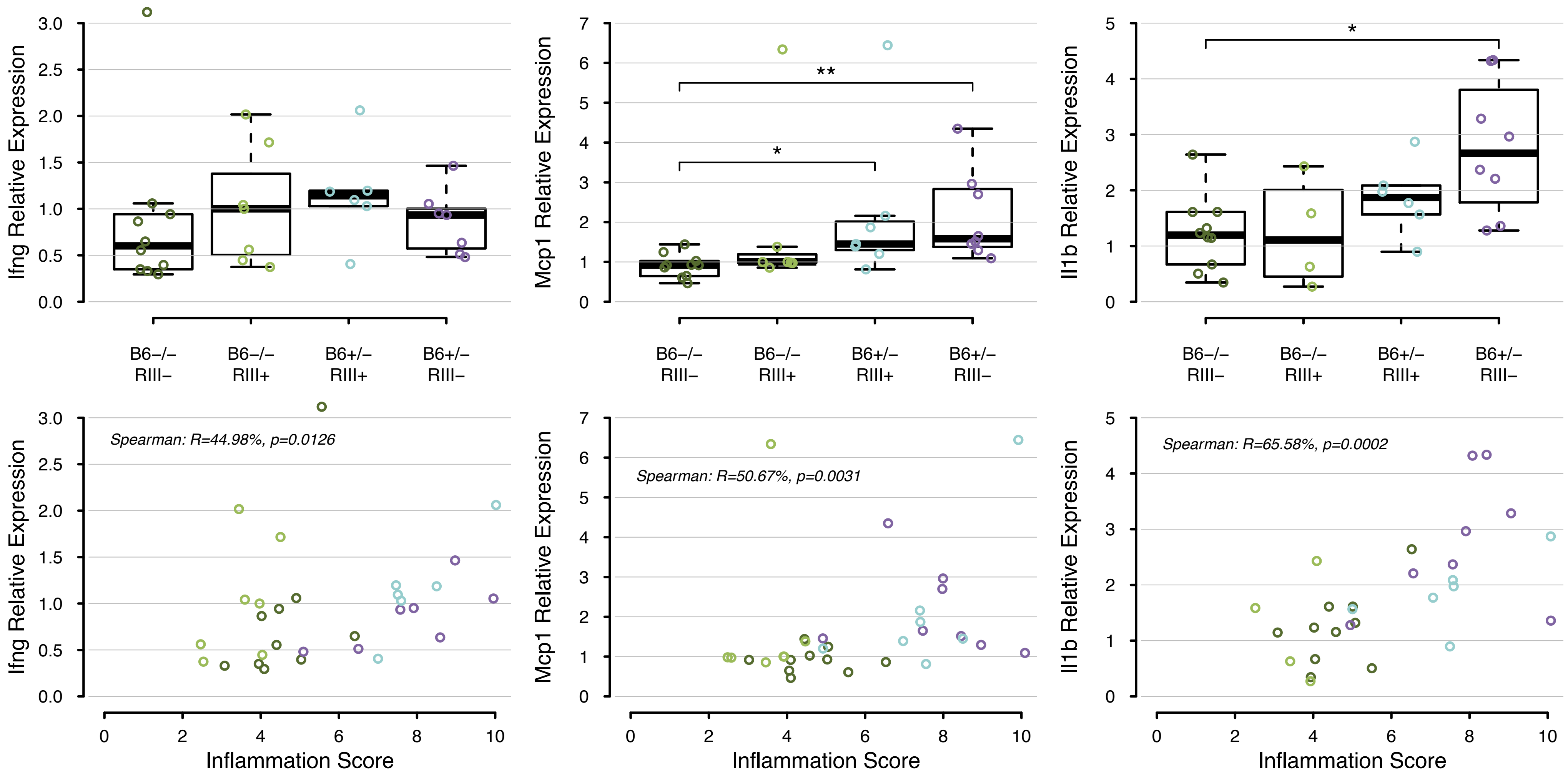


**Supplementary Figure 7: Relative expression of immunity genes in laboratory mice infected with *Morganella*.** Relative expression of IFNg (left), MCP1 (center) and Il1b (right) according to *B4galnt2* genotype (top) and histology-based inflammation score (bottom) in laboratory mice infected with *Morganella*. Relative expression was calculated through the delta delta Ct method by normalization to the house-keeping gene HPRT1, and the mean detla Ct of the B6$^{-/-}$ RII$^{+}$ group. Colors in the bottom panels represent *B4galnt2* genotype as displayed in the top panels. Comparisons between genotype categories in the top panels were made using pairwise Wilcoxon tests with "FDR" correction for multiple testing; ** p<0.01, * p<0.05.

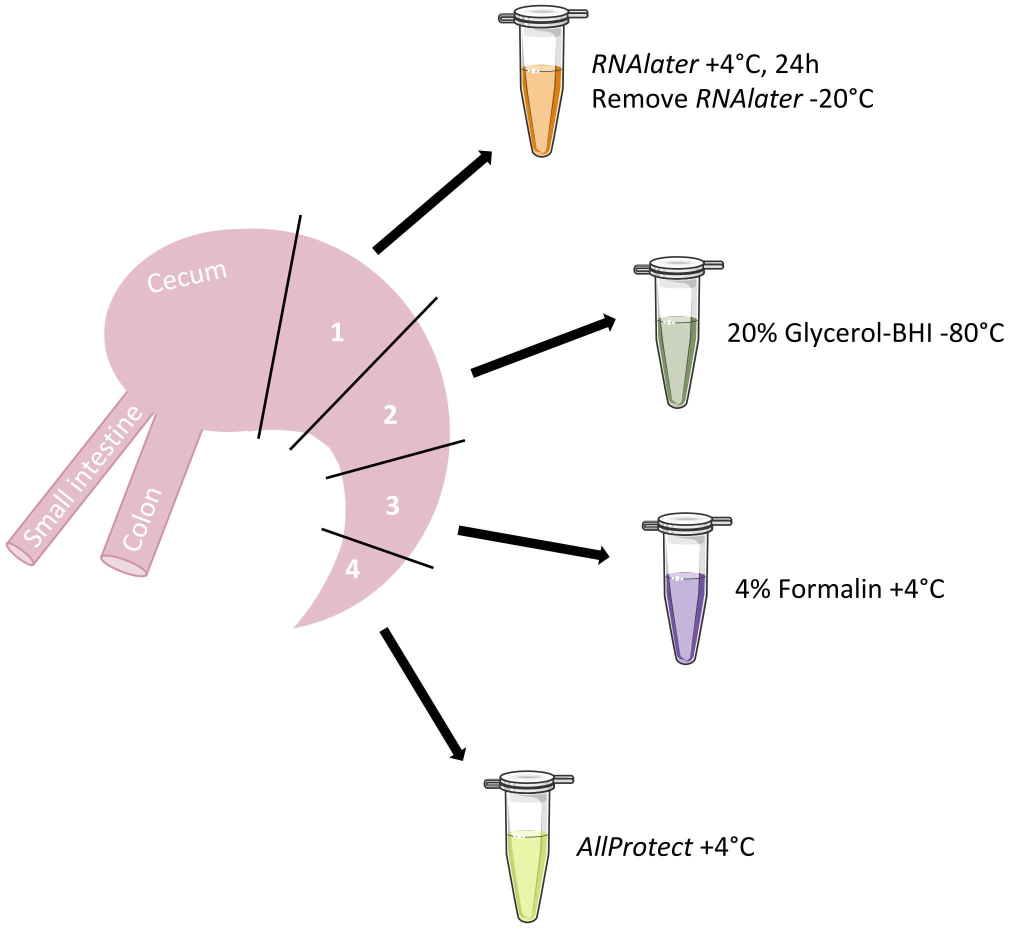


**Supplementary Figure 8: Sampling strategy.** Schematics of the sampling and storing of cecum samples from wild mice.
